# Histone Deacetylase Activity Modulates Alternative Splicing

**DOI:** 10.1371/journal.pone.0016727

**Published:** 2011-02-02

**Authors:** Jarmila Hnilicová, Samira Hozeifi, Eva Dušková, Jaroslav Icha, Tereza Tománková, David Staněk

**Affiliations:** Department of RNA Biology, Institute of Molecular Genetics AS CR, Prague, Czech Republic; Centre de Regulació Genòmica, Spain

## Abstract

There is increasing evidence to suggest that splicing decisions are largely made when the nascent RNA is still associated with chromatin. Here we demonstrate that activity of histone deacetylases (HDACs) influences splice site selection. Using splicing-sensitive microarrays, we identified ∼700 genes whose splicing was altered after HDAC inhibition. We provided evidence that HDAC inhibition induced histone H4 acetylation and increased RNA Polymerase II (Pol II) processivity along an alternatively spliced element. In addition, HDAC inhibition reduced co-transcriptional association of the splicing regulator SRp40 with the target fibronectin exon. We further showed that the depletion of HDAC1 had similar effect on fibronectin alternative splicing as global HDAC inhibition. Importantly, this effect was reversed upon expression of mouse HDAC1 but not a catalytically inactive mutant. These results provide a molecular insight into a complex modulation of splicing by HDACs and chromatin modifications.

## Introduction

Pre-mRNA splicing is an essential step in eukaryotic gene expression and its regulation vastly increases the coding potential of our genome. Splicing is catalyzed by the spliceosome which consists of spliceosomal small ribonucleoproteins (snRNPs) and additional splicing factors [1]. The critical step in splicing is intron recognition; this is achieved through the association of the splicing machinery with pre-mRNA via RNA-RNA and protein-RNA interactions. Interestingly, there is an increasing body of evidence suggesting that these interactions are not the only determinants of the splice-site definition [2].

There are many examples of a close coupling between transcription and splicing ([3], [4] reviewed in [5], [6]). Several splicing factors interact with RNA polymerase II (Pol II), which is important for their recruitment to pre-mRNA and through the combination of Pol II processivity and promoter identity, splice-site selection is influenced ([7], [8], [9], [10] reviewed in [11]). This regulation involves a co-transcriptional definition of splice-sites, spliceosome assembly and splicing [12], [13]. Indeed, the major regulators of splicing, snRNPs and SR proteins, are found at the site of active transcription [14], [15], [16], [17], [18], demonstrating that the splicing machinery assembles while the pre-mRNA is still associated with the DNA template. Such an observation suggests that chromatin modification might potentially play a regulatory role in splicing.

In yeast, the histone acetyltransferase found in the SAGA complex, Gcn5 is involved in co-transcriptional recruitment of the U2 snRNP [19]. In higher eukaryotes, the SWI/SNF chromatin remodeling complex associates with pre-mRNA and regulates alternative splicing of endogenous genes [20], [21] and treatment with histone deacetylase inhibitor trichostatin A (TSA) affects minigene alternative splicing [8]. Additionally, splicing factors interact directly with modified histones, although the significance of these interactions for splicing regulation remains unclear [22], [23]. Recently, genome-wide nucleosome mapping revealed that nucleosome localization correlates with exon positioning and may be involved in exon recognition [24], [25], [26], [27]. The role of nucleosome packing was supported by finding that siRNA-induced formation of heterochromatin influenced alternative splicing [28]. H3K36 tri-methylation differs at alternative and consecutive exons and affects alternative splicing through splicing factor recruitment [29], [30], [31], [32]. In addition, cell membrane depolarization resulted in altered RNA polymerase II transcription and chromatin modifications, correlating with alternative splicing changes [30]. In this study we examined whether enzymes catalyzing histone deacetylation can modulate alternative splicing of human genes.

## Results

### HDAC activity regulates alternative splicing

**Figure 1:**
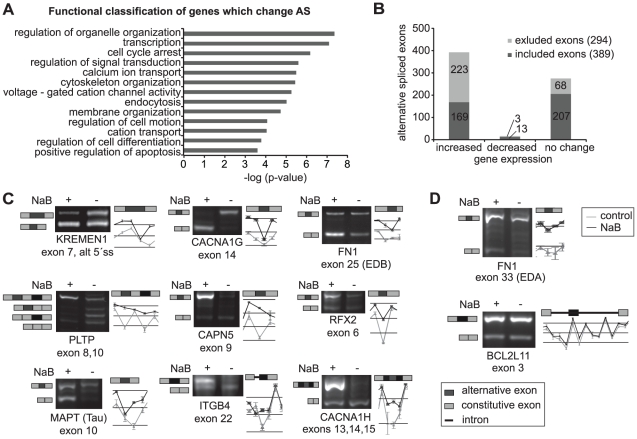
HDAC inhibition induces global changes in alternative splicing. HDAC activity was inhibited by treating cells with sodium butyrate (NaB) and splicing changes monitored by exon arrays. The splicing pattern of over 680 genes was changed (see Table S1). (A) Gene ontology analysis was performed with genes from the microarray annotation file as a background, enrichment score is the -log (p-value) of the chi-square test. Functional groups of over-represented genes with enrichment score >3 are shown. (B) Exons with high change (≥3-fold) were analyzed with respect to their inclusion or exclusion and divided into three groups according to the expression of a gene where the alternative exons are localized. (C) Several genes identified as top hits by microarray were confirmed by RT-PCR. Alternative exons were skipped after NaB treatment in three genes (*CACNA1G, FN1* –exon 24 and *MAPT).* In *KREMEN1* alternative 5′splice site was used and in *CAPN5, RFX2, ITGB4, PLTP* and *CACNA1H* alternative exons were included after HDAC inhibition. A graphic illustration of microarray data representing the same genomic loci as RT-PCR is shown next to the gels (non-treated cells - grey line; NaB treatment - black line). Exon array data show expression of individual alternative exons and neighboring constitutive exons. Relative decrease of the signal from alternative exon probes indicates alternative exon skipping, e.g. *CACNA1G* alternative exon signal from control cells (grey line) is higher compared to the surrounding constitutive exons than signal from NaB treated cells (black line). Although *CACNA1G* gene expression is elevated in NaB treated cells, the expression of alternative exon decreased, because this exon is preferentially skipped. (D) Two control exons that did not change splicing pattern upon NaB treatment.

In order to explore the effects of HDAC activity on alternative splicing we treated cells with the potent HDAC inhibitor, sodium butyrate (NaB) and monitored splicing changes by exon arrays. The analysis revealed that the splicing of 683 genes (out of 17,771 human genes included in the analysis) was altered upon HDAC inhibition (Table S1). Targeted genes are mainly involved in signaling (transmembrane transporters and receptors), transcription regulation, apoptosis, cell cycle and cell organization, all processes that regulate cell fate and differentiation (Fig. 1a). Interestingly, one of the target genes was encoding the Tau protein, which is abundantly expressed in central nervous system and enhanced inclusion of exon 10 causes neurodegenerative diseases as frontotemporal dementia with Parkinsonism linked to chromosome 17 (FTDP-17) [33]. The NaB treatment reduced expression of the splice variant that is upregulated during the disease (Fig. 1c). Exons with high splicing change (≥3 fold) were further analyzed with respect to their inclusion or exclusion (Fig. 1b). While we found a partial preference for overall exon inclusion (389 included/294 excluded) there was a strong correlation between increased gene expression and alternative events with a partial bias towards exon exclusion in up-regulated genes. Recently, a similar relationship was observed when a smaller set of genes was analyzed after UV irradiation [34]. These data suggest that HDAC inhibition did not only alter transcription but also substantially affected splicing pattern. To confirm exon-array results 16 target genes were further analyzed by conventional RT-PCR. Thirteen genes exhibited alternative splicing changes predicted by exon-arrays. Splicing pattern of nine of them is shown in Fig. 1c together with two control exons with no splicing change (Fig. 1d).

### Histone H4 acetylation correlates with alternative splicing

One of the genes most affected by HDAC inhibition was fibronectin (*FN1*). Given that fibronectin's alternative splicing variants are well described and have been extensively studied [35], [36], [37], we decided to use this gene to further characterize the role of HDACs in alternative splicing. As a model we analyzed exon 25 (called EDB or EDII) that was influenced by HDAC inhibition and exon 33 (called EDA or EDI) that did not alter splicing pattern upon NaB treatment. Moreover, one of the major advantageous is that proteins regulating splicing of the EDB exon were identified. It was shown that SR proteins, in particular SRp40, and PTB are important for EDB inclusion [38], [39], [40], [41].

**Figure 2:**
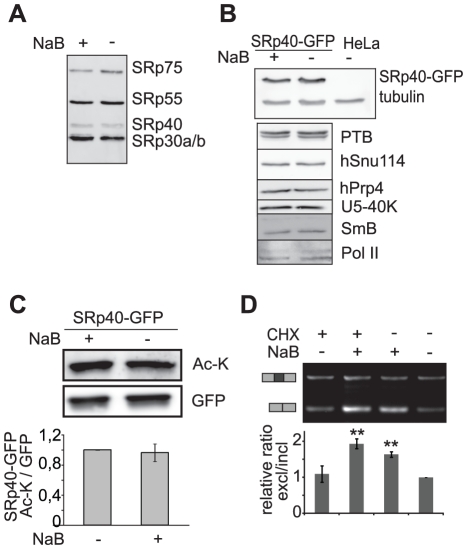
HDAC inhibition does not alter phosphorylation, acetylation or expression of SRp40. (A) HeLa cells or (B) HeLa cells stably expressing SRp40-GFP from BAC were treated for 15 h with NaB and levels of SR proteins were analyzed by Western blotting with the m104 antibody recognizing an SR protein phospho-epitope (A) or anti-GFP antibody (B). In addition, HDAC inhibition did not alter expression of several splicing proteins or RNA polymerase II. (C) SRp40-GFP was immunoprecipitated from SRp40-GFP stable cell line before and after HDAC inhibition and acetylation assayed by anti-acetyl lysine antibody. No significant change was observed after six hours NaB treatment. Representative Western blot and the average of three independent experiments is shown. (D) Inhibition of protein synthesis does not affect alternative splicing of the EDB exon in the *FN1* gene. Cells were treated for 9 h with cycloheximide (*CHX*), sodium butyrate (*NaB*) or both and EDB splicing assayed by RT-PCR and RT-qPCR (graph). Inhibition of HDAC by NaB had a similar effect on EDB skipping after CHX treatment. A representative RT-PCR (gel) and the average of three independent quantitative RT-PCR experiments (graph) are shown including SEM, ** indicates p≤0.01 of the t-test with respect to non-treated cells.

To test a potential mechanism via HDACs influence alternative splicing we first analyzed whether HDAC inhibition affected expression of general splicing proteins (namely snRNP specific proteins) and splicing regulators (SR proteins or PTB) (Fig. 2a,b). Using the monoclonal antibody m104 that recognizes a set of phosphorylated SR proteins including SRp40 [42] we showed that HDAC inhibition did not significantly alter the level of phosphorylated SR proteins (Fig. 2a). In addition, we used a HeLa cell line stably expressing SRp40-GFP from a bacterial artificial chromosome (BAC) that preserved endogenous SRp40 regulatory elements [43]. No change in SRp40-GFP expression was observed following the NaB treatment (Fig. 2b). Moreover, we did not observe any difference in expression of PTB, Pol II or core spliceosomal components hSnu114, hPrp4, U5-40K and SmB. It was shown recently that several splicing regulators including SRp40 are acetylated [44]. Therefore, we probed acetylation level of SRp40 before and after HDAC inhibition but did not find any significant changes in SRp40 acetylation status (Fig. 2c).

To further test whether the HDAC inhibition caused overexpression of other splicing factors that may potentially regulate fibronectin alternative splicing, cellular protein synthesis was inhibited in conjunction with HDAC activity. The inhibition of protein synthesis itself did not have any significant effect on EDB splicing. Further, we did not observe any differences in the splicing of the EDB exon whether cells were treated in combination with the ribosomal and HDAC inhibitors or HDAC inhibitor alone (Fig. 2d). These data indicated that NaB treatment did not influence splicing via altered expression or modification of regulatory proteins.

**Figure 3:**
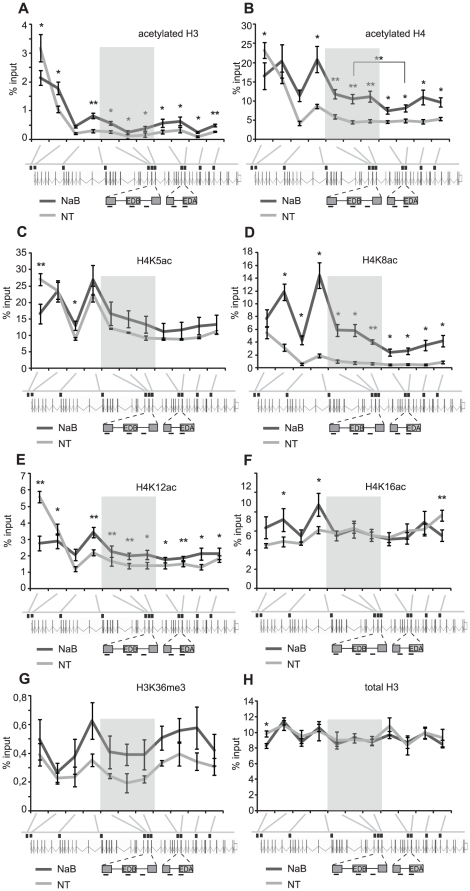
HDAC inhibition induces histone acetylation along the *FN1* gene. Chromatin immunoprecipitations were performed 6 h after HDAC inhibition with NaB using antibodies against (A) acetylated H3, (B) acetylated H4, (C) acetylated H4 lysine 5, (D) acetylated H4 lysine 8, (E) acetylated H4 lysine 12, and (F) acetylated H4 lysine 16. In addition, (G) tri-methylated lysine 36 histone H3, and (H) total H3 were probed. Probes detecting gene loci around alternative EDB exon are shaded. The average of at least three experiments is shown including SEM, ** indicates p≤0.01 and * p≤0.05 of the t-test.

Next, we performed a detailed analysis of chromatin marks along the *FN1* gene including the EDA exon where we did not detect any change in alternative splicing after NaB treatment. First we determined the level of acetylated histone H3 and H4. Acetylation of histone H3 was maximal at the promoter and dropped in the body of the gene. HDAC inhibition resulted in decreased H3 acetylation at the promoter (likely reflecting clearance of the promoter; see Fig. 3h) and partial increase within the gene (Fig. 3a). In non-treated cells general H4 acetylation was highest at the promoter. After HDAC inhibition, general H4 acetylation was more uniform and high all over the gene (Fig. 3b). Interestingly, the increase of H4 acetylation was significantly higher at the EDB exon than at the EDA exon, whose splicing did not respond to HDAC inhibition. Notably, different lysine residues within histone H4 contributed differently to the general H4 acetylation with highest increase at the lysines 8 and 12 (Fig. 3c–f). Next, we analyzed the level of lysine 36 tri-methylation at histone H3 as this modification was recently described as a marker of exon associated chromatin that influenced alternative splicing [30], [31], [32]. H3K36 tri-methylation increased partially within the gene body, including the alternative exon (Fig. 3g). At the same time no change was found in general nucleosome occupancy within the body of the gene (Fig. 3h). Together, these data show that HDAC inhibition has a global effect on chromatin modifications within the *FN1*.

**Figure 4:**
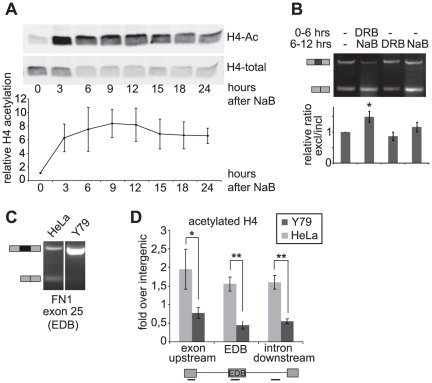
H4 acetylation correlates with EDB exon skipping. (A) The dynamics of histone H4 acetylation was assayed by Western blotting at different times after NaB treatment. A representative blot and the average of three experiments are shown including SEM. (B) EDB splicing was assayed six hours after HDAC inhibition. To reduce the possible effects of mRNA synthesis before HDAC inhibition, cells were treated with the Pol II inhibitor, DRB before the addition of HDAC inhibitor (*NaB*). The same amount of total RNA was used in each reaction. Following Pol II inhibition changes in EDB splicing were detected six hours post NaB treatment. A representative RT-PCR (gel) and the average of three independent quantitative RT-PCR experiments (graph) are shown including SEM. (C) EDB inclusion was analyzed in two different cell lines (Hela and Y79) using RT-PCR. (D) Histone H4 acetylation in HeLa and Y79 was assayed by chromatin immunoprecipitation at the EDB exon and the surrounding regions (see graphical representation of the gene loci below the graph). The average of three experiments is shown including SEM, ** indicates p≤0.01 and * p≤0.05 of the t-test.

As the maximal changes observed were in the acetylation of histone H4, we tested whether this modification correlated with alternative splicing. First, we measured the dynamics of H4 acetylation after HDAC inhibition in HeLa cells (Fig. 4a). Histone acetylation rapidly increased after 3 h of treatment and reached maximal levels within 6–9 h. However, little splicing effects were observed within this time period (Fig. 4b and data not shown). This discrepancy could be caused by mRNA being synthesized and spliced before HDAC inhibition. Thus, the presence of this mRNA might delay the detection of any splicing changes. To reduce the effect of mRNA spliced before HDAC inhibition, cells were incubated for 6 h with DRB, a reversible inhibitor of Pol II. Following this incubation, the Pol II inhibitor was removed and the cells were treated with NaB for an additional 6 h and EDB inclusion analyzed (Fig. 4b). The subsequent results show that as soon as 6 h post HDAC inhibition, the splicing of *de novo* synthesized pre-mRNA was altered. To further test the correlation between histone H4 acetylation and *FN1* splicing, we analyzed H4 acetylation in a retinoblastoma derived cell line Y79 that almost exclusively included the alternative exon (Fig. 4c). Using three different loci of the fibronectin gene, we show that general histone H4 acetylation is reduced in Y79 cells with respect to HeLa cells (Fig. 4d).

### Pol II processivity changes after HDAC inhibition

**Figure 5:**
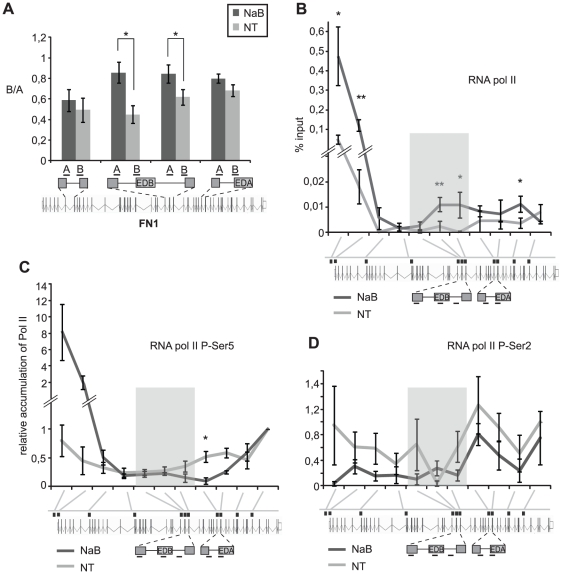
Pol II processivity correlates with exon skipping. (A) Pol II processivity was determined as a ratio of two pre-mRNA sequences (B:A) along several regions of the *FN1* gene. The abundance of given pre-mRNA locus was determined by quantitative PCR. The increased ratio after NaB treatment indicates higher Pol II processivity over the assayed region. Pol II exhibited high processivity increased significantly upstream and downstream of the EDB exon but not over consecutively spliced intron or alternative EDA exon that was not affected by NaB treatment. The average of three experiments is shown including SEM, * indicates p≤0.05 the t-test. (B-D) HDAC inhibition affects Pol II distribution. HDAC activity was inhibited by NaB for six hours and distribution of (B) total Pol II, (C) Pol II phosphorylated at the C-terminal domain Ser-5, and (D) Pol II phosphorylated at the C-terminal domain Ser-2 was assayed along the gene by chromatin immunoprecipitation. Probes detecting gene loci around alternative EDB exon are shaded. The tested gene loci are the same as in fig. 3. The average of three experiments is shown including SEM, ** indicates p≤0.01 and * p≤0.05 of the t-test.

Our data suggest a link between alternative splicing and H4 acetylation. It was previously shown that H4 acetylation was associated with Pol II processivity [45]. Moreover, there have been several observations that suggested a close relationship between Pol II dynamics and alternative splicing [7], [46]. Based on these observations we decided to analyze whether HDAC inhibition affects Pol II processivity. We probed Pol II processivity at several *FN1* gene loci by measuring the ratio between two pre-mRNA fragments as described previously [30] (Fig. 5a). Our results showed the highest increase of Pol II processivity in proximity of the EDB exon that was excluded but only small change at the alternative EDA exon that did not change splicing after HDAC inhibition.

Next, we determined the distribution of total Pol II as well as Pol II phophorylated at the C-terminal domain along the fibronectin gene after HDAC inhibition (Fig. 5b–d). Consistent with elevated levels of fibronectin mRNA (∼5-fold, see Table S1), we observed an increased occupancy of total Pol II and Pol II phosphorylated at Ser-5 at the promoter indicating a higher frequency of transcription initiation (Fig. 5b,c). Surprisingly, we found lower Ser-2 phosphorylation along the gene upon HDAC inhibition with EDB exon as the only exception, which likely reflects accumulation of total Pol II at the EDB exon (Fig. 5d). In addition, we observed accumulation of total Pol II at the EDB exon and the intronic sequence downstream of the EDB exon. Together with measurements of Pol II processivity these data indicated that HDAC inhibition resulted in increased Pol II processivity at upstream and downstream introns and slow down at the EDB exon. HDAC inhibition thus had a complex effect on Pol II distribution, phosphorylation and processivity along the fibronectin gene.

### HDAC inhibition decreases the association of SRp40 with the *FN1* gene

**Figure 6:**
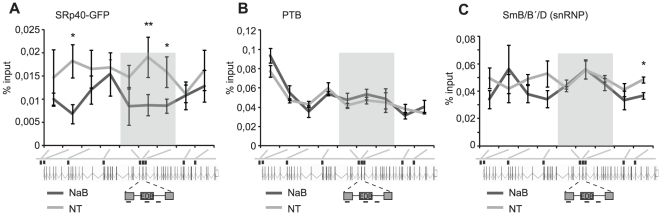
HDAC inhibition reduces SRp40 association with the EDB exon. (A) SRp40-GFP stably expressed from a BAC cell line was immunoprecipitated using an anti-GFP antibody. SRp40 interaction with the *FN1* gene was measured in cells treated for 6 h with NaB and in control cells. No changes in distribution of another splicing regulator PTB (B) or general splicing factors Sm proteins (C) were detected. Probes detecting gene loci around alternative EDB exon are shaded. The average of 3-5 experiments is shown, including SEM, ** indicates p≤0.01 and * p≤0.05 of the t-test.

The EDB exon is regulated by several *cis-*regulatory elements. A couple SRp40 binding sites were found within the EDB exon and the downstream intron, while PTB sites were identified upstream of the alternative exon. It was reported that EDB exon is recognized and spliced co-transcriptionaly [12] and that SR proteins directly associate with the nascent RNA at the transcription site [17]. Therefore, we decided to test whether HDAC inhibition influenced the interaction of SRp40 or PTB with the nascent RNA at the transcription site. To detect SRp40 at the transcription unit, we used a recently developed system that utilizes GFP tagged SR proteins and chromatin immunoprecipitation (ChIP) [17]. This report showed that GFP tagged SR proteins are expressed at the same or at lower levels than the endogenous protein and that the behavior of this tagged variant is indistinguishable from its endogenous counterpart. Our results revealed that SRp40 association with the *FN1* gene decreased upon HDAC inhibition, with the most significant decrease at the alternative EDB exon (Fig. 6a). The observed decrease was not a result of overall reduction of SRp40 protein level because HDAC inhibition did not alter SRp40 expression (Fig. 2b). In contrast, we did not detect any significant changes in association of PTB or general splicing Sm proteins over the alternative EDB exon indicating that changes in SRp40 interaction were not due to general changes in gene accessibility (Fig. 6b,c). These data indicated that HDAC activity influenced co-transcriptional association of SRp40 with the EDB exon.

### HDAC1 activity modulates fibronectin alternative splicing

**Figure 7:**
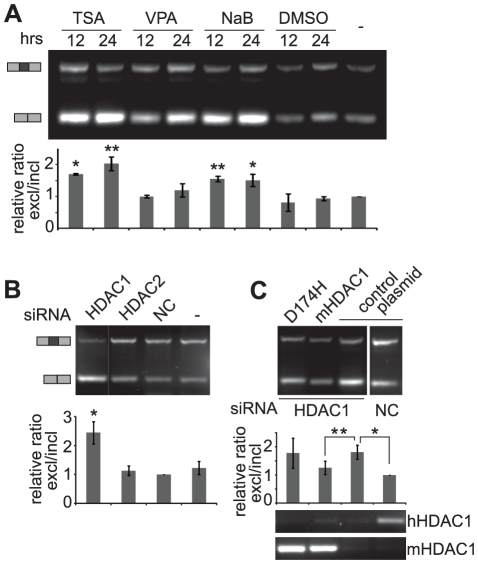
HDAC1 depletion affects EDB splicing. (A) Cells were treated with three different HDAC inhibitors (trichostatin A - TSA, valproic acid - VPA and sodium butyrate - NaB) for 12 h and 24 h. Their effect on fibronectin alternative EDB exon splicing was analyzed by RT-PCR (gel) or quantitative RT-PCR (graph). DMSO and non-treated cells served as negative controls. (B) HDAC1 and HDAC2 were down-regulated using siRNA and the resulting EDB splicing assayed. Depletion of HDAC1 but not HDAC2 resulted in EDB exon skipping. For knock-down efficiency see Fig. S1. (C) Endogenous HDAC1 was knocked-down by siRNA. The EDB skipping phenotype was rescued by expressing the mouse HDAC1 homologue (*mHDAC1*) but not an inactive mouse HDAC1 mutant (*D174H*). *NC* - negative control siRNA, *control plasmid* - GFP transfected cells. Expression of endogenous human (*hHDAC1*) and ectopically expressed mouse HDAC1 (*mHDAC1*) is shown below the graph. Graphs represent the average of three independent experiments measured by quantitative RT-PCR including SEM, ** indicates p≤0.01 and * p≤0.05 of the t-test.

To test whether the observed effects on alternative splicing were specific for NaB or whether it was a general property of HDAC inhibitors we reduced HDAC activity using three different inhibitors - TSA, valproic acid (VPA) and NaB and determined their effect on splicing of the fibronectin exon EDB. TSA is a pan-HDAC isoform inhibitor that *in vitro* displays nM potency against HDAC classes I (HDAC 1, 2, 3 and 8) and II (HDAC 4, 5, 6, 7, 9 and 10) with the exception of HDAC8, which is in the low µM range [47]. VPA is an established drug in the long-term therapy of epilepsy. It is a class I selective HDAC inhibitor, which *in vitro* inhibits HDAC 1, 2, 3 and 8 in the mM range [48]. Finally, sodium butyrate, a short-chain fatty acid sodium salt, primarily inhibits class I HDACs, but also HDAC 10 [47]. Following the treatment of cells with each HDAC inhibitor we observed the exclusion of the EDB exon in all cases, albeit with differing efficiencies, which might reflect the fact that targets of these inhibitors were not fully overlapping (Fig. 7a). The effect on alternative splicing was detectable after 12 h of HDAC inhibition and sustained for an additional 12 h. The smallest exclusion was observed after VPA treatment and Western blot analysis of H4 acetylation revealed that VPA had the smallest effect on H4 acetylation (data not shown). These results correlated with previous finding that different inhibitors regulated acetylation of core histones with different efficiencies [44].

To further investigate the role of HDACs in alternative splicing, two highly expressed enzymes from the HDAC class I family, HDAC 1 and 2, were knocked-down by RNAi (Fig. S1). Similar to HDAC inhibition, depletion of HDAC1 but not HDAC2 had a strong effect on fibronectin splicing, resulting in EDB skipping (Fig. 7b). To demonstrate the splicing effect of the RNAi treatment was specific to HDAC1, we used RNAi-resistant mouse HDAC1, which reverted the EDB splicing pattern (Fig. 7c and Fig. S1a). The requirement for HDAC1 activity was further supported by expression of HDAC1 mutant carrying an inactivating mutation within the active site [49], which was unable to rescue the splicing phenotype after RNAi treatment. These results together with HDAC inhibition data demonstrate that the enzymatic activity of HDAC1 is important for the regulation of fibronectin alternative splicing.

## Discussion

There is an increasing amount of evidence to show that chromatin modifications play a vital role in pre-mRNA splicing. However, little is known about the mechanism and factors involved in this coupling. In this study, we show that alternative splicing of a hundreds of genes is regulated by HDAC activity. Recently published *in vitro* data indicate that several HDAC inhibitors work by stalling the splicing reaction, suggesting that protein acetylation might have a role in regulating splicing activity. However, Kuhn and his co-workers did not identify a potential protein target, leaving the mechanism of HDAC inhibitor action on splicing open [50]. In our study, we did not observe any decrease in global mRNA production indicating that general splicing was not inhibited after HDAC inhibition with NaB. Thus, it remains to be clarified whether the observed *in vitro* effects of HDAC inhibitors apply to *in vivo* as well. Interestingly, about half of the proteins found in the spliceosome are acetylated but the functional role of these acetylations in splicing is yet to be established [44]. In addition, Mathias Mann and his colleagues tested two different HDAC inhibitors (SAHA targeting both HDAC classes I and II and MS-275 targeting class I) and found surprisingly low overlap of proteins with enhanced acetylation (43 out of 1750 proteins). Interestingly, histones were among the common targets and their acetylation increased after treatment with both drugs [44]. Here, we showed that at least two different inhibitors had the same effect on fibronectin splicing suggesting that both act via increased acetylation of histones as shared targets of different HDAC inhibitors. Moreover, we analyzed acetylation of the splicing regulator SRp40 but did not detect any changes upon HDAC inhibition. Despite that we cannot absolutely rule out the possibility that HDACs affect splicing via deacetylation of splicing proteins.

Recently it was shown in yeast that histone acetyltransferase Gcn5 affects co-transcriptional recruitment of U2 snRNPs [19]. The authors proposed that H3 acetylation at the promoter is responsible for the association of splicing factors with Pol II, resulting in their recruitment to the transcription unit. Our results with HDAC inhibitors suggest that histone acetylation can play a more direct role within the gene body. Histone acetylation around alternatively spliced elements affects splicing through the alteration of Pol II processivity that consequently modulates co-transcriptional association of splicing regulators with the nascent RNA.

The coupling between the elongation rate and alternative splicing was previously indicated by several studies. It was shown that the mutation in Pol II affecting the speed of synthesis, introduction of a pause site into the DNA template or depletion of transcription elongation factors influence alternative splicing outcome of transiently expressed reporter genes [7], [11], [28], [30], [46], [51], [52]. However, a direct molecular link between transcription dynamics and alternative splicing has not been described. Karla Neugebauer and her colleagues showed that reduction of RNA synthesis rate by camptothecin increases association of general splicing factors with the transcription unit indicating a kinetic coupling between RNA synthesis and interaction of splicing factors with the nascent RNA [16]. Here we describe that increase of Pol II dynamics in the vicinity of the alternative EDB exon correlates with reduced co-transcriptional recruitment of SRp40 supporting the model of kinetic coupling between transcription and splicing. It was shown that co-transcriptional association of SR proteins with the transcription unit is RNA dependent and determined by the RNA recognition domain [17]. Here we show that SR protein interaction with the transcription unit also depends on HDAC activity. In addition, SRp40 distribution does not correlate with Pol II occupancy, indicating that SRp40 does not interact primarily with Pol II.

More than 20 years ago it was hypothesized that splice-site selection is accomplished within a short window after transcription when RNA is naked and splicing factors can interact with target sequences [53]. Since then, co-transcriptional recognition of splice-sites was further supported by the study of Roberts et al. [52] and the significance of co-transcriptional splice-site recognition was recently demonstrated using the EDB exon that is recognized and spliced co-transcriptionally [12], [13]. Here we propose that co-transcriptional recruitment of splicing factor is modulated by histone modifications and Pol II processivity, which provides a link between chromatin modifications, transcription and splicing. Because NaB treatment leads to exclusion as well as inclusion of alternative exons we speculate that HDAC inhibition reduces the interaction of splicing enhancers (in the case of exon exclusion) or silencers (in the case of exon inclusion).

Our data also provide an example of how global changes in chromatin structure can result in local changes within specific genes. Global analysis of alternative splicing further revealed that many genes whose alternative splicing is regulated by HDACs belong to families of proteins that are involved in signaling and cell organization; processes that are tightly connected with cell differentiation. The dynamic interplay between chromatin structure, transcription and splicing might explain why some genes are alternatively spliced in different cell types that contain the same or a similar set of splicing regulators and as such might represent an efficient method for global splicing regulation during development and cell differentiation. In addition, many genes affected by HDAC inhibitor are involved in regulation of membrane potential, a process linked to signal transduction in neurons. Thus, HDAC inhibitors (e.g. VPA) used in epilepsy treatment might act via modulation of splicing pattern in patient neurons. For example, misbalanced splicing of Tau protein is a primary cause of frontotemporal dementias. Interestingly, Tau gene is on the list of genes whose splicing is modulated by HDAC inhibition. HDAC inhibitors thus might serve as a drug that acts via alteration of splicing pattern, which can increase their therapeutic potential.

## Materials and Methods

### Cell culture and treatments

HeLa and HeLa-GFP-SRp40 cells were cultured in DMEM supplemented with 10% fetal calf serum, penicillin and streptomycin (Invitrogen) and treated with 50 µM DRB (5,6-Dichlorobenzimidazole 1-β-D-ribofuranoside), 5 mM sodium butyrate, 5 mM valproic acid, 330 nM trichostatin A (all from Sigma) or cycloheximide (50 µg/ml, Calbiochem). Y79 human retinoblastoma cells (ATCC HTB-18, a kind gift of Martina Zikova, IMG ASCR) were cultured in RPMI with 10% fetal calf serum, penicillin and streptomycin. HeLa-GFP-SRp40 cell line was a gift from Ina Poser and Tony Hyman from (Max Planck Institute of Molecular Cell Biology and Genetics, Germany).

### Antibodies

The anti-RNA polymerase II H5 monoclonal antibody (recognizing phosphoserine 2) and H14 antibody (recognizing phosphoserine 5) were purchased from Covance, polyclonal antibodies against acetylated lysine 16 histone H4, Pol II (clone H-224) and mouse anti-GFP antibody (used for Western blotting) were from Santa Cruz. Monoclonal antibodies specific for HDAC1, polyclonal for acetyl-histone H4, acetyl-histone H3, H4K5ac H4K8ac and H4K12ac were all purchased from Upstate. Anti-H3, anti-H3K36me3 and 8WG16 anti-RNA polymerase II antibody (used for chromatin immunoprecipitations), anti-HDAC2 and anti-PTB antibodies were purchased from Abcam. hSnu114, hPrp4 and U5-40K antiserum was a gift from R. Lührmann (Max Planck Institute of Biophysical Chemistry, Göttingen, Germany), monoclonal m104 antibody recognizing phosphorylated SR proteins was a gift from K. Neugebauer (Max Planck Institute of Molecular Cell Biology and Genetics, Germany), anti-tubulin antibody was kindly provided by Pavel Draber (Institute of Molecular Genetics ASCR, Prague, Czech Republic), goat anti-GFP antibody used for ChIP was received from David Drechsel (Max Planck Institute of Molecular Cell Biology and Genetics, Germany) and the monoclonal anti-PTB antibody (BB7, used for chromatin immunoprecipitations) was provided by Douglas L. Black (Howard Hughes Medical Institute, Los Angeles, USA). The anti-Sm antibody Y12 was produced from a hybridoma cell line (a gift of Karla Neugebauer, Max Planck Institute for Molecular Cell Biology, Dresden, Germany; [54]) in the antibody facility at the Institute of Molecular Genetics ASCR. The polyclonal pan anti-acetyl lysine antibody was obtained from Immunechem. Nonspecific mouse IgG and anti-mouse IgM were both purchased from Sigma.

### Plasmids and siRNA transfection

Mouse HDAC1 cDNA (gift form Konstantinos Anastassiadis, TUD, Dresden, Germany) was subcloned into EGFP-N1 with SalI/NotI. The D174H mutation was introduced by PCR site-directed mutagenesis as described previously [55]. List of primers used in this study is listed in List S1. Plasmids were transfected with FuGENE HD (Roche Applied Science) according to manufacture's protocol. Preannealed siRNA duplexes were obtained from Ambion: HDAC1 5′-GGGAUACUUUUAUGCAACCtt-3′; HDAC2 5′-GCCACUGCCGAAGAAAUGAtt-3′; The negative control 1 siRNA from Ambion was used as a negative control. Oligofectamine (Invitrogen) was used for siRNA transfection according to the manufacture's protocol. Cells were incubated for 48 h (78 h for rescue experiments) before further treatment.

### RNA isolation and RT PCR

Total RNA was purified with TRIzol (Invitrogen), reverse transcribed using SuperScript III (Invitrogen) and cDNA amplified by Taq polymerase (MBI Fermentas). Primers used for RT-PCR and qPCR are listed in List S1.

### Quantitative real-time PCR

The ratio of mRNA with EDB exon skipped/included was calculated from relative Ct values of primers 41196 (*FN1* exon 24) and 42658 (*FN1* exon 25, EDB) according to R_treatment_ = 2^(CtEDB – CtEDBupstream)^ and normalized to control cells (R = R_treated_/R_control_). RNA polymerase II processivity was calculated from relative Ct values of primer pairs A (upstream) and B (downstream) according to pre-mRNA ratio_distal/proximal_ = 2^(CtA – CtB)^.

### Exon arrays

HeLa cells were treated with 5 mM NaB for 15 h and their RNA was isolated with TRIzol. For each sample, 1 µg of total RNA was processed, amplified, and labeled according to the Affymetrix GeneChip Whole Transcript (WT) Sense Target Labeling Assay (P/N 701880 Rev. 5). This protocol resulted in biotinylated sense strand cDNA samples, which were subsequently hybridized to GeneChip Human Exon 1.0 ST Array (Affymetrix, Inc., U.S.). Washing, staining, and scanning of the arrays was done according to the Affymetrix GeneChip Expression Wash, Stain, and Scan User Manual (P/N 702731) protocol. The data were analysed with the Partek Genomics Suite 6.4 software (Partek Incorporated, U.S.) using the RMA (Robust Multi-Array) algorithm. Only probesets that were present in the ‘core’ meta-probe list (17 800 RefSeq genes and full-length GenBank mRNAs) were used to identify alternative splicing events with Alt-splice ANOVA. A list of genes with alternative splicing was generated by using alternative splicing p-values corresponding to the 0.005 FDR criterion as a cutoff. To identify differentially expressed genes a p-value of 0.05 FDR was used as a cutoff.

Changes in exon expression were normalized to the changes in gene expression (NaB_exon expression_/NT_exon expression_)/(NaB_gene expression_/NT_gene expression_) and all exons with values ≥3 were considered as included, exons ≤1/3 as excluded. All data are MIAME compliant and that the raw data has been deposited in a MIAME compliant database (GEO, the accession numbers is GSE17397) available at the Gene Expression Omnibus (GEO) (http://www.ncbi.nlm.nih.gov/geo/info/linking.html).

### ChIP assays

Y79 or Hela cells (treated with 5 mM NaB for 6 h) were washed with PBS, crosslinked with 1% formaldehyde/PBS for 15 min at room temperature and the reaction was stopped by the addition of glycine (final conc. 125 mM). Cells were scraped into RIPA buffer (150 mM NaCl, 1% NP-40, 0.5% deoxycholate, 0.1% SDS, 50 mM Tris-HCl, pH 8.0, 5 mM EDTA, 0.5 mM PMSF, complete protease inhibitor cocktail (Calbiochem), 50 mM NaF and 0.2 mM sodium orthovanadate) and sonicated to generate ∼500 nt chromatin fragments. The same total amount of protein (1 mg or 0.3 mg for histone H3) was used for immunoprecipitation. Immunoprecipitation with the appropriate antibodies (20 µg anti-P-ser 2 and anti-P-ser 5 RNA Pol II, 5 µg anti-RNA pol II, 12 µg goat anti-GFP, 5 µl anti-PTB, 500 µl Y12 cell supernatant and 3 µg anti-H3 per reaction) was performed at 4°C overnight. Subsequently, the beads were rinsed twice with RIPA, four times with 100 mM Tris-HCl, pH 8.5, 500 mM LiCL, 1% Nonidet P-40, 1% deoxycholic acid, twice again with RIPA and twice with TE. Protein-DNA complexes were eluted with 1% SDS for 10 minutes at 65°C, decrosslinked in the presence of 200 mM NaCl for 5 h at 65°C and treated with 20 µg proteinase K for 30 min at 45°C. DNA was phenol/chloroform extracted, precipitated and amplified by quantitative real-time PCR on a LightCycler 480 System (Roche Applied Science). Data sets were normalized to ChIP input values, and the relative proportions of gene fragments obtained from ChIP with a nonspecific antibody were subtracted from the values obtained from templates derived from ChIP with the specific antibody: 2^(Ct(input) – Ct(spec))^ -2^(Ct(input) – Ct(unspec))^. To measure the level of phosphorylated RNA pol II the signal obtained for gene regions was further normalized to the value obtained in non-treated cells with the primer pair *FN1* 65438. To compare the level of acetylated histone H4 on *FN1* in HeLa and Y79 cells the signal obtained for *FN1* was normalized to the primer pair amplifying an intergenic region on chromosome 10 where no annotated genes could be found [16].

### Native ChIP assays

Hela cells (treated with 5 mM NaB for 6 h) were scraped into PBS and resuspended in 0.3 M sucrose, 60 mM KCl, 15 mM NaCl, 5 mM MgCl_2_, 0.1 mM EGTA, 0,2% NP-40, 15 mM Tris-HCl, pH 7.7, 0.5 mM DTT, complete protease inhibitor cocktail (Calbiochem) and 5 mM NaB. Nuclei were released by passage through a 22 G needle and loaded on a sucrose gradient (1.2 M sucrose, 60 mM KCl, 15 mM NaCl, 5 mM MgCl_2_, 0.1 mM EGTA, 15 mM Tris-HCl, pH 7.7, 0.5 mM DTT, protease inhibitors and 5 mM NaB) and centrifuged for 20 min at 2000 g, 4°C. Pellets were resuspended in Mnase digestion buffer (0.32 M sucrose, 1 mM CaCl_2_, 4 mM MgCl_2_, 15 mM Tris-HCl pH 7.7 and protease inhibitors) and digestion performed for 6 min at 37°C (1U Mnase/30 µg chromatin). Reactions were stopped by EDTA (final concentration 10 mM) and centrifuged. The supernatant was taken and the pellet resuspended in 0.2 mM EDTA, 1 mM Tris/HCl, pH 7.7, incubated for 1 h at 4°C, centrifuged again and both supernatants mixed. ∼100 µg of chromatin was diluted in nChIP buffer (50 mM NaCl, 5 mM EDTA, 50 mM Tris/HCl, pH 7.7) and incubated overnight at 4°C with appropriate antibody (10 µg anti-acetyl H3, 6 µg anti-H3K36me3, 4 µg anti-H4K16ac, 10 µg nonspecific IgG, 5 µl anti-acetyl H4, 6 µl anti-H4K12ac and 5 µl anti-H4K5ac and anti-H4K8ac). The beads were washed once with nChIP buffer, then twice in the same buffer with increasing salt concentration (75 mM NaCl, 125 mM NaCl, 175 mM NaCl). Complexes were eluted with 1% SDS for 15 min at room temperature, treated with 20 µg proteinase K for 30 minutes at 45°C and DNA was recovered with the QIAGEN PCR Purification Kit, quantified by qPCR and signal compared to the input and non-specific antibody: 2^(Ct(input) – Ct(spec))^ -2^(Ct(input) – Ct(unspec))^.

### Western blotting and immunoprecipitation

Western blotting and immunoprecipitation was performed as described previously [55]. Protein extraction from TRIzol after RNA isolation was done according to manufacturer's protocol.

## Supporting Information

Figure S1 HDAC1 activity regulates fibronectin alternative splicing. (A) Before cells were transfected with wild type mouse HDAC1 or a control plasmid, endogenous HDAC1 was knocked-down by siRNA. Subsequently, EDB splicing was analyzed by RT-PCR. Expression of HDAC1 after siRNA treatment was assayed by Western blotting. The anti-HDAC1 antibody cross-reacted with mouse HDAC1, which resulted in an increased signal in the last line. Snu114 served as a loading control. (B) Reduction of HDAC2 expression after siRNA treatment. Total RNA and proteins were isolated from cells treated with HDAC2 siRNA, negative control siRNA or untreated. The relative amount of HDAC2 mRNA was assayed by RT-qPCR and protein by Western blotting.
(EPS)

List S1 Supplementary primer list.
(DOC)

Table S1 Alternative spliced genes decreased expression.
(XLS)
