# Supplementary material for: Histone Deacetylase Activity Modulates Alternative Splicing
Source: PLoS One. 2011 Feb 2;6(2):e16727. doi: 10.1371/journal.pone.0016727 (PMC3032741; doi:10.1371/journal.pone.0016727)
Supplement: List S1 — Supplementary primer list. (DOC) [file pone.0016727.s002.doc]

**Primers for RT PCR validation of alternative splicing:**

BCL2L11 exon 3 5´- CTTGCCAGGCCTTCAACCACTAT- 3´

5´- ACACCAGGCGGACAATGTAACGTA -3´

CACNA1G exon 14 5´- ATTTCATTGCCCTCATGACC -3´

5´- GCCAAGCACTTCTTCCTGTC -5´

CACNA1H exons 13-15 5´- ACAACGTGGCTACCTTCTGC- 3´

5´- TCCAGGAATGGTGAGCTCTT- 3´

CAPN5 exon 9 5´- GGAGGAGTGGCAGAAAGTGA -3´

5´- GCTGGATGCAGATCAGGACT -3´

FN1 (EDA) 5´- AATCCAAGCGGAGAGAGTCA-3´

5´- TTCATTGGTCCGGTCTTCTC -3´

FN1 exon 25 (EDB) 5´- AGTTGGTTAAATCAATGGATG -3 for reverse transcription

5´- TGGAGTACAATGTCAGTGTTT -3´

PCR

5´- CTGGACCAATGTTGGTGAATC -3´

ITGB4 exon 22 5´- ACCTGCTGAAGCCTGACACT -3´

5´- TGGTCTGCAGTGAGGGTGTA -3´

KREMEN1 exon 7 5´- GGAGCTTCTGGATGGCTACA -3´

5´- GGCTGTGACTGTGAGGATGA -3´

PLTP exon 8,10 5´- ATCTGCCCTGTCCTCTACCA -3´

5´- ACAGAGATGGTGGTGCCAGA -3´

RFX2 exon 6 5´- ATGGACAGCACCAGACACTC -3´

5´- TGGGTCTAGCTTGTGCTCCT -3´

MAPT exon 10 5´- AAGATCGGCTCCACTGAGAA -3´

5´- ATGAGCCACACTTGGAGGTC -3´

**Primers for quantitative PCR – chromatin immunoprecipitation:**

*FN1* promoter (-505) 5´- TTGATGACCGCAAAGGAAAC -3´

5´- TCGCAGCGAACAAAAGAGAT -3´

*FN1* exon 1 (48) 5´- CCGTCTCAACATGCTTAGGG -3´

5´- ATTTGCTGAGCCTGCCTCTT -3´

*FN1* exon 7 (10557) 5´- ATTAGGATCTGGCCCCTTCA -3´

5´- TGTGACACAGTGGCCATAGG -3´

*FN1* intron 14 - exon 15 (26036) 5´- AAAATGATGTTGGCGACGAG -3´

5´- CGTCTCTCCTGTCACGGTGT -3´

*FN1* exon 24 (upstream EDB, 41196) 5´- GGAAGAAGTGGTCCATGCTG -3´

5´- GGGACACTTTCCTTGTCATCC -3´

*FN1* exon 25 (EDB, 42658) 5´- AGGTGCCCCAACTCACTGACC -3´

5´- TGCCGCAACTACTGTGATGCGGTA -3´

*FN1* intron 25 (43389) 5´- GGGTAGAGTGGATGGGCATT -3´

5´- CATGCTTGTCCCCAGACTGT -3´

*FN1* exon 32 (upstream EDA) – intron 32, 53556 5´ - CAGCCCACAGTGGAGTATGT -3´

5´- GCAGTGGTTACGTACTGGTTACTG -3´

*FN1* intron 32 – exon 33 (EDA, 54761) 5´- TTTGCCTAACAGACATTGATCG -3´

5´- TGGAAACTTGCCCCTGTG -3´

*FN1* exon 38 (60499) 5´- CACCCAATTCCTTGCTGGTA -3´

5´- GGACCACTTCTCTGGGAGGA -3´

*FN1* exon 42 (65438) 5´- ACCAGTGCCACTCTGACAGG -3´

5´- TTCCCGAACCTTATGCCTCT -3´

intergenic 5´- GGCTAATCCTCTATGGGAGTCTGTC -3´ Chromosome 10, contig AL392045

region 5´- CCAGGTGCTCAAGGTCAACATC -3'

**Primers for quantitative PCR – RNA polymerase II speed (Fig. 4):**

*FN1* exon 6 – intron 6, **A** 5´- GAAACCTGCTCCAGTGCATC -3´

5´- CTCGTCCTGTGCCTCACC -3´

*FN1* intron 6 – exon 7, **B** 5´- TTTCATGCCATTAGGATCTGG -3´

5´- TGTGACACAGTGGCCATAGG -3´

*FN1* exon 24 – intron 24, **A** 5´- CACTGTCAAGGATGACAAGGAA -3´

5´- CCCCACTCTTATTGGAAGTGTC -3´

*FN1* intron 24 – exon 25 (EDB), **B** 5´- TTTTTCCCTCTATTTTCCTTTTG -3´

5´- GTTATATCAACAAAGCTTAGGTCAGTG -3´

*FN1* intron 25 (43389), **A** 5´- GGGTAGAGTGGATGGGCATT -3´

5´- CATGCTTGTCCCCAGACTGT -3´

*FN1* intron 25 – exon 26, **B** 5´- CCTTTCCAGCTACTTCGTTAGC -3´

5´- AATGTTGGTGAATCGCAGGT -3´

*FN1* exon 32– intron 32, **A** 5´ - CAGCCCACAGTGGAGTATGT -3´

5´- GCAGTGGTTACGTACTGGTTACTG -3´

*FN1* intron 32 – exon 33 (EDA) , **B** 5´- TTTGCCTAACAGACATTGATCG -3´

5´- TGGAAACTTGCCCCTGTG -3´

**Primers used for mouse HDAC1 mutagenesis and RT PCR:**

D174H 5´- GGGTGCTCTATATTCACATTGATATTCACC -3´

5´- GGTGAATATCAATGTGAATATAGAGCACCC- 3´

Mouse HDAC1 5´- GGAGAAGCCAGAAGCCAAAGGGGT-3´

5´- CTGAGAAGTGAGGAACTTGGGG-3´

Human HDAC1 5´- GGAGAAGCCAGAAGCCAAAGGGGT-3´

5´- GGAAGAAACGTGAGGGACTCAGCA-3´

**Primers for quantitative PCR – HDAC2 mRNA level**

*HDAC2* 5´- CGCATGACCCATAACTTGCT -3´

5´- ATTTCTTCGGCAGTGGCTTT-3´

18S rRNA 5´- TTGTTGGTTTTCGGAACTGAG -3´

5´- GCAAATGCTTCGGCTCTGGCT -3´
